# Protein Quality and Protein Digestibility of Vegetable Creams Reformulated with Microalgae Inclusion

**DOI:** 10.3390/foods12122395

**Published:** 2023-06-16

**Authors:** Barbara Prandi, Fatma Boukid, Simon Van De Walle, Sara Cutroneo, Josep Comaposada, Geert Van Royen, Stefano Sforza, Tullia Tedeschi, Massimo Castellari

**Affiliations:** 1 Department of Food and Drug, University of Parma, Parco Area delle Scienze 27/A, 43121 Parma, Italy; barbara.prandi@unipr.it (B.P.); sara.cutroneo@unipr.it (S.C.); stefano.sforza@unipr.it (S.S.); tullia.tedeschi@unipr.it (T.T.); 2 Institute of Agriculture and Food Research and Technology (IRTA), Food Industry Area, Finca Camps i Armet s/n, 17121 Girona, Spain; josep.comaposada@irta.cat (J.C.); massimo.castellari@irta.cat (M.C.); 3 ClonBio Group Ltd., 6 Fitzwilliam Pl, D02 XE61 Dublin, Ireland; 4 Flanders Research Institute for Agriculture, Fisheries and Food (ILVO), Brusselsesteenweg 370, 9090 Melle, Belgium; simon.vandewalle@ilvo.vlaanderen.be (S.V.D.W.); geert.vanroyen@ilvo.vlaanderen.be (G.V.R.)

**Keywords:** amino acids, protein in vitro digestibility, microalgae, protein degree of hydrolysis, cell walls

## Abstract

Microalgae are considered a valuable source of proteins that are used to enhance the nutritional value of foods. In this study, a standard vegetable cream recipe was reformulated through the addition of single-cell ingredients from *Arthrospira platensis* (spirulina), *Chlorella vulgaris*, *Tetraselmis chui*, or *Nannochloropsis oceanica* at two levels of addition (1.5% and 3.0%). The impact of microalgae species and an addition level on the amino acid profile and protein in vitro digestibility of the vegetable creams was investigated. The addition of microalgae to vegetable creams improved the protein content and the amino acid nutritional profile of vegetable creams, whereas no significant differences were observed in protein digestibility, regardless of the species and level of addition, indicating a similar degree of protein digestibility in microalgae species despite differences in their protein content and amino acid profile. This study indicates that the incorporation of microalgae is a feasible strategy to increase the protein content and nutritional quality of foods.

## 1. Introduction

Microalgae, including cyanobacteria, have a long history of use as a human food for their nutritional and environmental merits [1,2]. Microalgae can contribute to at least sixteen out of seventeen of the 2030 agenda of Sustainable Development Goals (SDGs; 2015–2030) [3]. This is due to their carbon dioxide (CO_2_)-fixing ability (10–50 times more than other terrestrial plants) to produce their own energy for growth, their limited need for land and water resources, their fast growth rate compared to terrestrial crops, and their ability to grow under different environmental conditions [4,5,6]. From a nutritional perspective, microalgae can be considered a source of high-value ingredients, such as proteins and bioactive peptides, pigments, antioxidants, vitamins, and omega-3 fatty acids, known for their beneficial health effects (e.g., anti-inflammatories, disease prevention, and metabolic alterations) [7,8]. The continuous advances in biotechnology boost the commercial exploitation of microalgae production in different sectors, such aquaculture, food, and feed, owing to higher productivity and better control of quality [9,10].

In the food industry, the application of microalgae is steadily increasing due to consumers’ awareness of the above-mentioned advantages [11,12,13]. The most-well known microalgae are *Arthrospira* spp. ‘Spirulina’ and *Chlorella* spp., and they are widely used in different types of products, e.g., couscous, vegetable creams, pasta, crackers, and other baked products [14,15,16]. These two species are approved for human consumption in Europe. *Tetraselmis chui* was also approved as a novel food according to Regulation (EC) No 258/1997 in 2017 [17], yet it is still used to a limited extent in food products.

In the frame of alternative proteins, microalgae are gaining momentum due to their high protein quantity (with a range from 30 to 70% of the dry mass) and quality (having a complete essential amino acid pattern) [18,19]. The protein content and composition of microalgae are closely related to several factors such as species, strain, growth stage, and culture conditions, such as temperature, medium composition, and light [20,21,22]. Protein digestibility is also a crucial parameter used to estimate protein quality. Protein digestibility refers to the amount of protein available for absorption after its digestion and can be assessed through in vitro or in vivo models [23]. In vitro static and dynamic models are most frequently used to simulate the digestion process due to their simplicity, low cost, and the fact that they do not include any ethical restrictions [23]. One of the most used in vitro methods is the harmonized INFOGEST in vitro digestion method.

Research studies reported different values of microalgae protein digestibility due to the use of different digestion models, species, cultivation methods, cell wall compositions, and cell disruption methods [24,25]. There is strong evidence suggesting that microalgae have poor protein digestibility since proteins are entrapped inside rigid and complex cell walls, limiting their contact with the digestive enzymes [26,27,28]. Furthermore, once microalgae are included in food, the digestibility of their protein fraction may vary depending on the specie, the level of addition, and the interaction with other food matrix components. Most studies focusing on the digestibility of food enriched with microalgae were conducted on bread and bakery products, while less interest was attributed to liquid or semi-solid foods. In this light, this study aimed to gather knowledge on the protein digestibility of vegetable creams enriched with microalgae. For this reason, four microalgal species (*A. platensis*, *C. vulgaris*, *T. chui*, and *N. oceanica*) were considered. First, total amino acid profile and the digestibility of microalgal single-cell ingredients were evaluated. Then, these ingredients were included in vegetable cream formulations to assess the effects of the species and the addition level on protein composition and digestibility.

## 2. Material and Methods

### 2.1. Material and Preparation Procedure of Vegetable Creams

For this study, dried single-cell ingredients of microalgae produced in closed photobioreactors were provided by Necton (Olhão, Portugal) and Allmicroalgae (Pataias, Portugal). The chemical composition of the microalgae used is reported in Table S1. These microalgae were used in the reformulation of a standard vegetable cream (STD). The list of ingredients and the preparation procedure of vegetable creams were previously reported [29]. In brief, 67.6% frozen vegetables (spinach, zucchini, chickpea, leek, broccoli, and chard bought from Geland, Girona, Spain), 29% mineral water, 2.9% sunflower oil, and 0.5% salt (purchased from a local supermarket) were used to prepare the standard vegetable cream formulation (STD). For microalgae-enriched vegetable creams, 4 microalgal species (*A. platensis*, *C. vulgaris*, *T. chui*, and *N. oceanica*) were included at two levels of addition (1.5 and 3.0%) that were calculated based on the total weight of the vegetable ingredients by the proportional retrieval from each vegetable. A total of 9 formulations were obtained [STD: control; SP1.5: *A. platensis* 1.5% (*w*/*w*); CV1.5: *C. vulgaris* 1.5% (*w*/*w*); TC1.5: *T. chui* 1.5% (*w*/*w*); NO1.5: *N. oceanica* 1.5% (*w*/*w*); SP3: *A. platensis* 3% (*w*/*w*); CV3: *C. vulgaris* 3% (*w*/*w*); TC3: *T. chui* 3% (*w*/*w*); NO3: *N. oceanica* 3% (*w*/*w*)]. The chemical composition of vegetable creams is reported in Table S2.

For each formulation, three independent replicates (1 kg each) were produced under the same conditions. Ingredients were cooked in a cooking robot (Thermomix^®^, Vorwerk, Wuppertal, Germany) at 90 °C for 25 min with continuous mixing (300 rpm). The cooked product was homogenized, packaged (200 mL glass bottles), cooled down (until 20 °C), and autoclaved (Ilpra Systems, Mataró, Spain) at 116 °C/75 min/1.2 Bar.

### 2.2. In Vitro Simulated Digestion

Simulated gastrointestinal digestion was performed following the INFOGEST 2.0 method [30], except for the addition of gastric lipase, which was skipped. The initial amount of sample was 1 g. All samples were digested in duplicate in the presence of the respective control sample, which followed the same protocol but without the addition of enzymes.

### 2.3. Measurements and Analysis

#### 2.3.1. Optical Microscopy

Microalgae single-cell ingredients were diluted to a concentration of 0.3% (*w*/*w*) and then characterized using an optical microscope (Axio Scope.A1; Carl Zeiss Microscopy GmbH, Goettingen, Germany). For each microalga, at least five microscopic images for each sample were taken with a 20× magnification lens with a digital camera (AxioCam 503 color; Carl Zeiss, Göttingen, Germany) under the same conditions (luminance and contrast ratio).

#### 2.3.2. Total Amino Acids

The total composition of amino acids, apart from Trp, was determined for the microalgae single-cell ingredients and vegetable creams prior to digestion, starting with 2.6–3.0 g of creams [31,32].

For the determination of Trp, 1.5–2.0 g of cream was weighed in an 18 mL Pyrex with a Teflon-coated screw cap, and 4 mL of 4 N NaOH was added, along with 150 µL of α-methyl-tryptophan (50 mg/100 mL) as an internal standard. The samples were mixed and incubated in an oil bath at 100 °C for 6 h. The samples were allowed to cool to room temperature and were neutralized with 6 N HCl. Samples were stored at −20 °C until the day of analysis. Then, they were transferred to disposable plastic tubes and centrifuged at 3220× *g* for 45 min at 4 °C. The supernatant was transferred to a 10 mL flask, made up to the mark with distilled water, and filtered with 0.45 µm syringe filters. For quantification, the response factor was prepared by adding the standards (150 µL of tryptophan 50 mg/100 mL and 150 µL α-methyl-tryptophan 50 mg/100 mL) into a 10 mL flask and making it up to volume with distilled water. The samples and the response factor were injected directly into UPLC-MS (Waters, Milford, MA, USA) for analysis, according to these conditions: column (Waters, Milford, MA, USA): ACQUITY UPLC Protein BEH C18 (300 Å, 1.7 μm, 2.1 mm 170 × 150 mm) with ACQUITY UPLC Peptide BEH C18 VanGuard pre-column (130 Å, 1.7 17 μm, 2.1 mm × 5 mm); mobile phase: eluent A (Milli-Q H_2_O + 0.2% CH_3_CN + 0.1% HCOOH) and eluent B (CH_3_CN + 0.2% Milli-Q H_2_O + 0.1% HCOOH); gradient: 0–1.8 min isocratic 100% A, 1.8–13.2 min linear from 100% A to 50% A, 13.2–14 min 50% A, 14–14.5 min linear from 50% A to 0% A, 14.5–16.1 min 0% A, 16.1–16.5 min linear 0% A to 100% A, 16.5–29.5 min 100% A; acquisition mode: SIR (*m*/*z* 188.0; 202.1; 205.0; 219.1). All solvents and standards were purchased from the Sigma-Aldrich Chemical Co. (St. Louis, MO, USA).

The tryptophan content (g) was calculated by dividing the tryptophan area in the sample by the response factor (A_tryptophan_/A_α-methyltryptophan_ in the equimolar standard solution) and making a proportion to the amount of internal standard added (g) in the sample.

#### 2.3.3. Protein Content

The total protein content (TPC) was determined according to the Kjeldahl method (VELP Scientifica, Usmate, Italy). The TPC was determined by multiplying the value obtained by 6.25, a commonly used conversion factor, which allowed us to estimate the TPC starting from the total nitrogen content [33].

#### 2.3.4. Degree of Protein Hydrolysis (DH%)

The degree of hydrolysis is the percentage of free amino groups (presumably derived from the protein digestion process) with respect to the total amount of amino groups present (including the amino groups involved in the internal protein peptide bonds). The free amino groups were determined spectrophotometrically (340 nm) after the 5 µL reaction of the digested samples with 2.4 mL of *o*-pthaldialdehyde/*N*-acetylcysteine reagent, by interpolation with an external calibration curve made with isoleucine (0.125–0.25–0.5–1–2 mg/mL, in duplicate). The total nitrogen was divided by the average residual molecular weights of the amino acids to obtain the moles of peptide bonds [34]. The DH% was determined after digesting all samples (and their respective digestion controls) in duplicate. The absorbance of the blank (obtained by replacing the 5 µL of sample with distilled water) was subtracted from the absorbances of the samples and controls. Reagents and standards were purchased from the (Sigma-Aldrich Chemical Co. (St. Louis, MO, USA).

#### 2.3.5. Statistical Analysis

Significant differences among the microalgae species were assessed by using analysis of variance (ANOVA) with a confidence interval of 95% (*p* ≤ 0.05). Multivariate analysis of variance (MANOVA) was used to determine the effect of microalgae species (S) and the level of addition (LA) on the amino acid composition and the degree of hydrolysis of vegetable creams. MANOVA was performed based on fixed factors using Pillai’s trace test. The percentages of total variations were computed to determine the contribution of the factors (S and LA) and their interactions in the variance of each parameter. The percentage of total variation was computed to explain the variance of each parameter as a function of the sum squares of the main factors and their interaction. Significant differences among the mean values were calculated using Duncan’s test. All experimental data were statistically analyzed using SPSS version 19.0 (SPSS Inc., Chicago, IL, USA).

## 3. Results and Discussion

This study aimed to gather knowledge on the protein digestibility of vegetable creams enriched with microalgae. For this reason, four species of microalgae were considered (*A. platensis*, *C. vulgaris*, *T. chui* and *N. oceanica*). First, the total amino acid profile and digestibility of the powdered microalgae were assessed (Section 3.1). Then, these were included in vegetable cream formulations to evaluate the effects of the species and level of addition on the protein composition and digestibility of the creams (Section 3.2).

### 3.1. Microalgae Protein Characterization

#### 3.1.1. Appearance of Microalgal Cells

To obtain information about the structural characteristics of the microalgae ingredients, microscopic images were taken (Figure S1). *N oceanica* had small cells (2–5 µm, [35]) with a spherical shape and a smooth surface, where an eyespot was present in accordance with previous findings [35,36]. Cells were dispersed in the solution while keeping their intactness. On the other hand, the cells of *C. vulgaris* were agglomerated in morula-like structures (around 15 µm with cells of around 2 µm) [37], probably because they were spray dried (when small droplets containing cells are dried, cells stick together). The cells had a spherical shape, and the walls seemed intact. In *A. platensis*, cells were aggregated into small filaments. In untreated cells (fresh biomass), filaments were longer and tended to form spirals [38]. This underlined that this microalga was more sensitive to thermal and physical treatments because of its less thick cell walls compared to *C. vulgaris* [39,40,41]. *T. chui* had ovoid or ellipsoidal cells and flattened equatorially. It seemed that the cells remained intact owing to their rigid cell walls, as previously reported [42]. Overall, cells of the four microalgae species maintained their cell structure, except for *A. platensis*, showing short and not elongated filaments.

#### 3.1.2. Protein Content and Amino Acid Composition

Protein content was first determined by the Kjeldahl method (Table S1) and was analyzed using SDS page (Figure S2), as reported in the experimental section. As reported in Table S1, the highest protein content was found in *A. platensis* (57.50 g/100 g dry weight), which was consistent with studies reporting a range of variability from 50 to 70% [43,44]. *C. vulgaris* showed a lower value (26.30 g/100 g dry weight) than that reported in the literature (up to 60%) [45]. This is probably due to the differences in medium composition, strain, and cultivation conditions. The protein content of *T. chui* (45.26 g/100 g dry weight) was again found consistent with previous works (≈35–40/100 g dry weight) [42] and within the same range was compared to *N. oceanica* (45.00 g/100 g dry weight) (9previously reported to be ≈29–48 g/100 g dry weight; [46,47]). These microalgae (except *A. platensis*) have protein contents higher or within the same range of most plant protein sources, such as oat, chickpea, soybean, lupine, and fava bean (30–40 g/100 g dry weight) [48,49,50].

Knowledge of amino acid (AA) composition (with particular reference to essential amino acids, EAAs) of microalgae has great importance in the establishment of their nutritional value and potential uses in food applications [51]. According to the different total protein content, the amino acid content of the ingredients from the four microalgae species included in this study (Table 1) differed significantly, except for cysteine. In *C. vulgaris* and *A. platensis,* total amino acids did not differ significantly from crude protein measured by the Kjeldahl method, indicating that the analyzed samples have a negligible amount of non-protein nitrogen. However, the protein contents (by Kjeldahl) of *N. oceanica* and *T. chui* were significantly higher than total amino acids, indicating that the samples analyzed have non-protein nitrogen*. A. platensis* had the highest amino acid content, followed by *N. oceanica*, *T. chui*, and *C. vulgaris*. Essential and non-essential amino acids followed the same pattern as total amino acids. The most abundant amino acids in all microalgae species were Arg, Asp, Ala, Lys, and Glu, followed by Pro, Leu, Gly, and Ser. These results agree with previous studies [51,52,53], even if other authors reported higher amino acid amounts, comparable to those of eggs and soybeans [54,55]. These differences in values can be due to different harvesting regimes and seasons, drying processes, and strains [56,57].

#### 3.1.3. Protein in Vitro Digestibility

**Figure 1:**
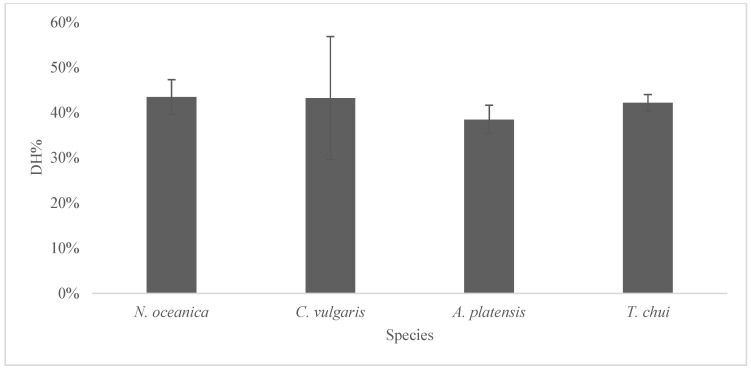
The degree of hydrolysis of microalgae single-cell ingredients after in vitro digestion (mean values ± std dev).

The average DH% value of microalgae powders after simulated digestion was 42% in a range of 29–57% (Figure 1), indicating that approximately half of the peptide bonds were broken after the digestion process. This aligns with a previous study on *A. platensis* (dry powder digested using serine endopeptidase) that showed an average value DH% around 55% [58]. Similarly, the DH% of *N. oceanica* was around 54% (a range of variability of 48–59%) [59]. However, in another study where the microalgae biomasses’ in vitro digestibility was evaluated by the method of Boisen and Fernández (using pepsin and pancreatin) [60], *T. chui*, *C. vulgaris*, and *A. platensis* were reported to have higher values (85%, 81%, and 76%, respectively) [61]. These differences can be due to the protocols used to assess digestibility and to the fact that the authors digested a fresh microalgae biomass (not subject a drying step). Higher digestibility for *A. platensis*, *C. vulgaris*, and *N. chui* (88%, 87%, and 85%) was also reported by [62]. Higher values (up to 94%) were obtained for *C. vulgaris* and *A. platensis* when digested using the method of Boisen and Fernández [60]. The digestibility of the same *C. vulgaris* biomass was assessed with three different methods, and significantly different results were found (84%, 80%, and 70%) [63]. Furthermore, quantification methods differ among studies, and this impacts the results. Overall, the high variability in microalgae protein digestibility found in the literature is probably due to multiple factors, as digestion models, sample processing, enzyme mixture, and differences related to microalgae biomass such as specie/strain, cultivation methods, and the degree of cell wall disruption [59,62].

Under our conditions, microalgae ingredients from the four species showed a similar DH% (sig. 0.662 in the ANOVA). Other studies showed higher protein digestibility in the case of ingredients from *A. platensis*, having a less robust cell wall made of four layers of fibrils and peptidoglycan [39,64], while microalgae with thick cells walls, such as *N. oceanica*, *C. vulgaris*, and *T. chui*, provided lower protein digestibility values due to their limited accessibility for the digestive enzymes [39,40,41].

### 3.2. Effect of Microalgae Incorporation on Protein Composition of Vegetable Creams

Total protein content of vegetable creams differed among the species showing that SP3 (Table S2) had the highest value, and it increased significantly as a function of the increased level of addition. Consistently, this addition significantly increased the total amount of amino acids (Table 2), which was proportional to the quantity of microalgae added, regardless of species. The inclusion of *A. platensis* caused the highest increase of the total amino acid content (AA) in the vegetable creams, from 13.57% (% of dry weight) for the standard cream to 16.78% in the product enriched with 3.0% of single-cell ingredients (SP3). This can be attributed to the fact that *A. platensis* had the highest initial amount of protein (57.50% of dry biomass) (Table S1). As per the single AA, serine, lysine, histidine, phenylalanine, arginine, methionine, and tryptophane did not show any significant variations as a function of the formulation. On the other hand, glycine, alanine, proline, valine, threonine, isoleucine, leucine, aspartic acid, glutamic acid, tyrosine, and cysteine showed significant increases due to the addition of microalgae in vegetable cream formulations.

Based on Pillai’s trace test of MANOVA, microalgae species and their level of addition had a significant impact on the AA profile, while no significant effect of the interaction was found. Table 3 shows the contribution of each factor to the variance of each amino acid. AA, NAA, and EAA were influenced by the microalgae species and, mainly, by the level of addition. The same trend was followed by Val, Thr, Ile, Leu, and Glu. Gly and Ala were found to be almost equally controlled by the species and the level of addition. Pro was mainly governed by the species, while the level of addition did not show a significant effect. On the other hand, the level of addition and the species did not cause any significant effect on the levels of the amino acids Ser, Lys, Phe, Arg, Met, and Trp.

The nutritional value of the different formulations was calculated, taking as reference the requirement of essential amino acids set by the FAO (Table 4). Even the reformulated vegetable creams rather lacked in essential amino acids compared to the needs of children from birth to 6 months of age; Ile, Lys, and Trp were decidedly scarce in almost all the preparations tested, with very low scores especially for Trp. The situation improved considering the needs of children from 6 months to 3 years, for whom the quantity of Ile is sufficient and only Trp and Lys are limiting amino acids, with scores included between 0.58 and 0.82 (Lys) and 0.64 and 0.97 (Trp). Finally, considering the needs of older children, adolescents, and adults, the most limiting amino acid is mainly Lys, with scores included between 0.68 and 0.97 (Lys), in line with those of plant-based proteins. In all cases, the addition of microalgae increases the amino acid score; therefore, according to recent studies [65], microalgae are potential alternatives of high-quality proteins and amino acids to complement conventional sources. Among the tested formulations, the amino acid profiles of CV3 and NO3 are perfectly suitable from the nutritional point of view for older children, adolescents, and adults.

### 3.3. Effect of Microalgae Incorporation on Protein Digestibility of Vegetable Creams

**Figure 2:**
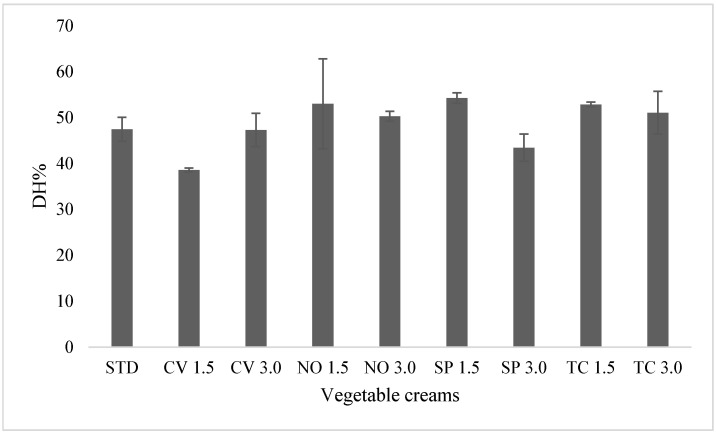
Degree of hydrolysis (DH%) of the different vegetable creams after in vitro digestion.

The protein digestibility (DH%) of vegetable creams was between 38 and 53% (Figure 2), indicating that between one third and one half of the peptide bonds are broken after the digestion process, which is also consistent with that observed for pure microalgae. No significant differences were found between the DH%s of the formulations (sig. 0.053 in the ANOVA). Species, addition level, and their interactions also did not affect the DH% (as from MANOVA). Therefore, the high protein digestibility of creams was not negatively affected by the microalgae addition. This can be due to the low amounts of fortification or/and to the protein digestibility of the microalgae ingredients, which is similar to that of the STD vegetable cream (average DH% value of microalgae of 42%, as reported in Section 3.1).

Other authors reported that adding 3% (*w*/*w*) *A. Platensis* to cookies, crackers, and “crostini” reduced the product protein digestibility [61,67,68,69]. The inclusion of *T. chui* in bread at higher levels (12% *w*/*w*) reduced the protein digestibility coefficient (56.1%), while 12% *C. vulgaris* reduced digestibility compared to the control bread (from 60.3% to 68.5%) [45]. Depending on the matrix, the protein digestibility of cookies made with *C. vulgaris* (6% *w*/*w*) was found to be similar to that of the control (70%), while the digestibility of crackers was significantly reduced after adding *C. vulgaris* [67,69]. In a recent study, the protein digestibility of couscous enriched with *C. vulgaris* (6% *w*/*w*) was significantly increased compared to the control, probably due to texture softness that might increase protein bioavailability [70]. Therefore, it seems that the impact of the reformulation with microalgae ingredients on the food protein digestibility can vary, and it is a complex phenomenon influenced by many variables, such as the type of microalgae ingredient (species and processing) but also by the type of food matrix, whose digestion can be unaffected, improved, or hindered [23]. In the present study, high protein digestibility was preserved (around 40–50%) independently from the level of protein addition and the species of microalgae.

## 4. Conclusions

Based on our results, microalgae single-cell ingredients differed in protein composition (protein content and amino acid profile). Nevertheless, protein digestibility was found to be similar among the studied species, despite their cell structure differences. The incorporation of microalgae in vegetable creams significantly increased protein content and total amino acids according to the microalgae specie and the level of addition. These results sustain the addition of microalgae to enhance the amino acid profile and boost the protein content of vegetable creams. Even more importantly, the protein digestibility of reformulated creams did not decrease if compared to the standard formulation (without microalgae). Further studies focusing on a higher level of microalgal addition would be of interest to explore their impact on protein nutritional quality and digestibility.

## Figures and Tables

**Table 1:** Amino acid composition (g/100 g dry weight) of the single-cell microalgae ingredients. Small letters indicate significant differences (*p* < 0.05) between means (n = 2 analyses) on the same line.

		CV	NO	SP	TC
**Gly**	***	1.85 ± 0.08 a	2.65 ± 0.01 c	3.29 ± 0.06 d	2.06 ± 0.03 b
**Ala**	***	2.36 ± 0.07	2.95 ± 0.12	4.12 ± 0.11	2.50 ± 0.06
**Ser**	***	1.18 ± 0.03 a	1.60 ± 0.01 b	2.57 ± 0.06 c	1.17 ± 0.05 a
**Pro**	***	2.09 ± 0.08 b	2.21 ± 0.02 c	2.43 ± 0.01 d	2.02 ± 0.02 a
**Val**	***	1.50 ± 0.09 a	2.50 ± 0.01 b	3.36 ± 0.15 c	1.75 ± 0.07 a
**Thr**	***	1.15 ± 0.03 a	2.23 ± 0.01 c	2.93 ± 0.04 d	1.68 ± 0.02 b
**Ile**	***	0.89 ± 0.07 a	1.88 ± 0.03 c	3.08 ± 0.14 d	1.20 ± 0.06 b
**Leu**	***	1.92 ± 0.07 a	3.20 ± 0.04 c	3.94 ± 0.11 d	2.30 ± 0.10 b
**Asp**	***	3.71 ± 0.14 a	4.93 ± 0.02 b	6.58 ± 0.10 c	5.16 ± 0.10 b
**Lys**	***	3.24 ± 0.11 c	2.67 ± 0.01 b	2.99 ± 0.22 b c	1.86 ± 0.01 a
**Glu**	***	3.51 ± 0.14 a	4.78 ± 0.03 b	8.46 ± 0.02 c	4.80 ± 0.08 b
**His**	***	0.58 ± 0.02 a	0.77 ± 0.01 b	0.88 ± 0.04 c	0.55 ± 0.01 a
**Phe**	***	0.94 ± 0.02 a	2.27 ± 0.23 c	2.61 ± 0.12 c	1.61 ± 0.06 b
**Arg**	***	2.71 ± 0.14 a	2.60 ± 0.01 a	3.98 ± 0.01 c	3.75 ± 0.01 b
**Tyr**	***	0.74 ± 0.03 a	1.32 ± 0.03 c	2.28 ± 0.02 d	0.95 ± 0.01 b
**Met**	***	0.65 ± 0.05 a	0.98 ± 0.01 c	1.39 ± 0.01 d	0.81 ± 0.06 b
**Cys**	ns	0.32 ± 0.01	0.26 ± 0.05	0.46 ± 0.15	0.48 ± 0.03
**EAA**	***	10.85 ± 0.21 a	16.50 ± 0.28 b	21.20 ± 0.80 c	11.75 ± 0.21 a
**NAA**	***	18.50 ± 0.70 a	23.30 ± 0.01 b	34.20 ± 0.10 c	22.90 ± 0.01 b
**AA**	***	29.35 ± 0.90 a	39.80 ± 0.20 c	55.35 ± 0.60 d	34.65 ± 0.20 b

*** = *p* < 0.001; ns = not significant; EAA = essential amino acids; NAA = non-essential amino acids; AA = total amino acids. Different letters in the same row indicate significant (*p* < 0.05) differences between samples for the given parameter.

**Table 2:** Amino acid composition (% on dry weight) of the analyzed vegetable creams (mean ± std dev).

	Significance	STD	CV1.5	NO1.5	SP1.5	TC1.5	CV3	NO3	SP3	TC3
**Gly**	*	0.61 ± 0.01	0.68 ± 0.03	0.62 ± 0.18	0.78 ± 0.01	0.74 ± 0.00	0.72 ± 0.01	0.80 ± 0.00	0.85 ± 0.01	0.81 ± 0.01
**Ala**	***	0.72 ± 0.03	0.82 ± 0.13	0.86 ± 0.04	0.96 ± 0.04	0.83 ± 0.02	0.94 ± 0.02	0.96 ± 0.01	1.14 ± 0.03	0.96 ± 0.03
**Ser**	ns	0.93 ± 0.03	0.93 ± 0.03	1.03 ± 0.19	1.01 ± 0.01	0.94 ± 0.02	0.92 ± 0.03	0.97 ± 0.01	1.09 ± 0.01	1.01 ± 0.02
**Pro**	**	0.73 ± 0.01	0.83 ± 0.06	0.82 ± 0.01	0.84 ± 0.02	0.81 ± 0.03	0.86 ± 0.02	0.87 ± 0.00	0.88 ± 0.02	0.86 ± 0.02
**Val**	**	0.64 ± 0.03	0.69 ± 0.03	0.71 ± 0.05	0.79 ± 0.05	0.74 ± 0.01	0.74 ± 0.02	0.75 ± 0.02	0.86 ± 0.03	0.74 ± 0.01
**Thr**	***	0.69 ± 0.02	0.74 ± 0.04	0.77 ± 0.07	0.82 ± 0.01	0.76 ± 0.02	0.76 ± 0.02	0.82 ± 0.01	0.94 ± 0.00	0.83 ± 0.00
**Ile**	***	0.53 ± 0.03	0.54 ± 0.01	0.57 ± 0.03	0.66 ± 0.05	0.58 ± 0.01	0.57 ± 0.03	0.61 ± 0.01	0.74 ± 0.03	0.59 ± 0.01
**Leu**	***	1.11 ± 0.04	1.14 ± 0.05	1.22 ± 0.03	1.33 ± 0.06	1.21 ± 0.02	1.22 ± 0.04	1.33 ± 0.01	1.43 ± 0.00	1.29 ± 0.03
**Asp**	*	1.53 ± 0.08	1.60 ± 0.28	1.83 ± 0.08	1.92 ± 0.04	1.69 ± 0.09	1.81 ± 0.08	1.92 ± 0.09	2.02 ± 0.03	1.98 ± 0.05
**Lys**	ns	0.59 ± 0.23	0.54 ± 0.18	0.49 ± 0.01	0.46 ± 0.05	0.42 ± 0.04	0.55 ± 0.01	0.54 ± 0.00	0.51 ± 0.02	0.52 ± 0.04
**Glu**	*	2.22 ± 0.06	2.42 ± 0.42	2.52 ± 0.05	2.75 ± 0.12	2.38 ± 0.10	2.52 ± 0.07	2.60 ± 0.09	2.97 ± 0.05	2.77 ± 0.02
**His**	ns	0.41 ± 0.12	0.38 ± 0.03	0.39 ± 0.06	0.37 ± 0.05	0.36 ± 0.01	0.24 ± 0.00	0.32 ± 0.01	0.35 ± 0.00	0.31 ± 0.03
**Phe**	ns	0.76 ± 0.01	0.77 ± 0.04	0.78 ± 0.05	0.86 ± 0.06	0.82 ± 0.00	0.79 ± 0.04	0.82 ± 0.04	0.85 ± 0.03	0.83 ± 0.03
**Arg**	ns	1.15 ± 0.55	0.90 ± 0.13	0.81 ± 0.12	0.89 ± 0.01	0.86 ± 0.02	0.87 ± 0.03	0.78 ± 0.01	0.93 ± 0.03	1.00 ± 0.09
**Tyr**	*	0.44 ± 0.01	0.46 ± 0.03	0.48 ± 0.05	0.57 ± 0.04	0.48 ± 0.00	0.46 ± 0.03	0.50 ± 0.02	0.58 ± 0.01	0.49 ± 0.03
**Met**	ns	0.27 ± 0.01	0.30 ± 0.01	0.31 ± 0.00	0.34 ± 0.01	0.29 ± 0.01	0.29 ± 0.01	0.33 ± 0.05	0.33 ± 0.04	0.37 ± 0.07
**Cys**	**	0.16 ± 0.01	0.23 ± 0.02	0.18 ± 0.02	0.18 ± 0.01	0.23 ± 0.00	0.19 ± 0.02	0.18 ± 0.01	0.22 ± 0.00	0.24 ± 0.01
**Trp**	ns	0.07 ± 0.00	0.07 ± 0.01	0.09 ± 0.02	0.09 ± 0.01	0.08 ± 0.01	0.08 ± 0.02	0.10 ± 0.01	0.09 ± 0.01	0.09 ± 0.01
**EAA**	**	5.07 ± 0.29	5.19 ± 0.28	5.33 ± 0.29	5.71 ± 0.10	5.27 ± 0.03	5.25 ± 0.17	5.62 ± 0.04	6.10 ± 0.08	5.58 ± 0.17
**NAA**	*	8.50 ± 0.42	8.86 ± 1.04	9.15 ± 0.71	9.91 ± 0.12	8.96 ± 0.23	9.29 ± 0.31	9.58 ± 0.20	10.68 ± 0.06	10.12 ± 0.01
**AA**	*	13.57 ± 0.72	14.06 ± 1.31	14.48 ± 1.00	15.62 ± 0.22	14.23 ± 0.26	14.54 ± 0.48	15.21 ± 0.23	16.78 ± 0.02	15.70 ± 0.15

* = *p* < 0.05; ** = *p* < 0.01; *** = *p* < 0.001; ns = not significant; EAA = essential amino acids; NAA = non-essential amino acids; AA = total amino acids.

**Table 3:** Multivariate analysis of the amino acid profiles of vegetable creams reformulated with different microalgae and two levels of addition. Percentages of sum squares (SS%) were calculated to determine the contribution of each factor in the variability of each AA.

	Microalgae Species (S)		Level of Addition (LA)		The Interaction (S × LA)	
	SS%	Significance	SS%	Significance	SS%	Significance
**Gly**	42	*	45	ns	13	ns
**Ala**	48	***	50	**	2	ns
**Ser**	3	ns	69	ns	28	ns
**Pro**	80	**	15	ns	5	ns
**Val**	19	*	75	**	6	ns
**Thr**	31	**	60	***	8	ns
**Ile**	10	*	84	***	6	ns
**Leu**	30	***	70	***	1	ns
**Asp**	39	*	51	ns	9	ns
**Lys**	37	ns	48	ns	16	ns
**Glu**	27	*	62	*	11	ns
**His**	59	*	16	ns	26	ns
**Phe**	7	ns	81	ns	12	ns
**Arg**	6	ns	64	ns	30	ns
**Tyr**	2	ns	98	**	0	ns
**Met**	17	ns	38	ns	45	ns
**Cys**	1	ns	68	**	31	ns
**Trp**	19	ns	78	ns	3	ns
**EAA**	21	*	75	**	5	ns
**NAA**	35	*	58	*	6	ns
**AA**	31	*	64	*	5	ns
**TPC**	22	***	75	***	2	***

EAA = essential amino acids; NAA = non-essential amino acids; AA = total amino acids; TPC: total protein content; S = species; LA = level of addition; S × LA = interaction between species and level of addition; ns: not significant; *: *p* ≤ 0.05; **: *p* ≤ 0.01; ***: *p* ≤ 0.001; SS: sum of squares.

**Table 4:** Amino acid scoring pattern of vegetable creams based on the essential amino acid requirements in the report of an FAO expert consultation [66]. SAA = sulfur amino acids; AAA = aromatic amino acids.

Age Group	His	Ile	Leu	Lys	SAA	AAA	Thr	Trp	Val
**Scoring Pattern mg/g Protein Requirement**									
**Infant (birth to 6 months)**	21	55	96	69	33	94	44	17	55
**Child (6 months to 3 year)**	20	32	66	57	27	52	31	8.5	43
**Older child, adolescent, adult**	16	30	61	48	23	41	25	6.6	40
	**Scoring pattern (birth to 6 months)**								
**Formulation**	**His**	**Ile**	**Leu**	**Lys**	**SAA**	**AAA**	**Thr**	Trp	Val
**STD**	1.44	0.71	0.85	0.63	0.95	0.94	1.16	0.32	0.86
**CV1.5**	1.43	0.77	0.93	0.61	1.24	1.02	1.31	0.34	0.98
**NO1.5**	1.47	0.83	1.02	0.57	1.18	1.07	1.40	0.43	1.04
**SP1.5**	1.38	0.94	1.09	0.53	1.24	1.19	1.47	0.40	1.13
**TC1.5**	1.36	0.84	0.99	0.48	1.24	1.09	1.35	0.37	1.05
**CV3**	0.97	0.87	1.07	0.67	1.23	1.12	1.45	0.39	1.13
**NO3**	1.32	0.96	1.20	0.68	1.36	1.21	1.62	0.49	1.18
**SP3**	1.43	1.15	1.27	0.63	1.41	1.29	1.81	0.45	1.33
**TC3**	1.23	0.89	1.13	0.63	1.53	1.17	1.58	0.44	1.13
	**Scoring pattern (child 6 months to 3 year)**								
**Formulation**	His	Ile	Leu	Lys	SAA	AAA	Thr	Trp	Val
**STD**	1.51	1.22	1.24	0.76	1.17	1.70	1.64	0.64	1.10
**CV1.5**	1.50	1.32	1.35	0.74	1.52	1.85	1.86	0.68	1.26
**NO1.5**	1.54	1.43	1.48	0.69	1.44	1.93	1.99	0.86	1.33
**SP1.5**	1.45	1.62	1.59	0.64	1.52	2.16	2.08	0.79	1.44
**TC1.5**	1.43	1.44	1.44	0.58	1.51	1.96	1.92	0.74	1.35
**CV3**	1.02	1.49	1.55	0.81	1.51	2.02	2.06	0.78	1.45
**NO3**	1.38	1.65	1.74	0.82	1.66	2.19	2.29	0.97	1.51
**SP3**	1.51	1.97	1.84	0.76	1.72	2.34	2.57	0.90	1.70
**TC3**	1.29	1.53	1.64	0.77	1.87	2.12	2.25	0.88	1.45
	**Scoring pattern (Older child, adolescent, adult)**								
**Formulation**	His	Ile	Leu	Lys	SAA	AAA	Thr	Trp	Val
**STD**	1.89	1.30	1.34	0.90	1.37	2.16	2.04	0.83	1.19
**CV1.5**	1.88	1.41	1.46	0.88	1.78	2.34	2.30	0.87	1.35
**NO1.5**	1.93	1.53	1.60	0.82	1.69	2.45	2.47	1.10	1.43
**SP1.5**	1.81	1.73	1.72	0.76	1.78	2.73	2.58	1.02	1.55
**TC1.5**	1.78	1.53	1.56	0.68	1.78	2.49	2.38	0.95	1.45
**CV3**	1.28	1.59	1.68	0.97	1.77	2.56	2.56	1.01	1.56
**NO3**	1.73	1.76	1.89	0.97	1.95	2.78	2.84	1.25	1.63
**SP3**	1.88	2.11	2.00	0.90	2.02	2.96	3.19	1.16	1.83
**TC3**	1.61	1.64	1.77	0.91	2.19	2.69	2.79	1.14	1.56

## Data Availability

Data is contained within the article or Supplementary Material.

## Supplementary Materials

The following supporting information can be downloaded at: https://www.mdpi.com/article/10.3390/foods12122395/s1, Figure S1: Microscopic images of microalgae single cell ingredients. Photos by *M. Soares* and *B. Schmid*; Figure S2: SDS-PAGE of spinach creams subjected to simulated gastrointestinal digestion (digested) or not (controls); Table S1: Chemical composition of microalgae powders (g/100 g); Table S2: Chemical composition of vegetable creams (g/100 g).
