# Supplementary material for: Protein Quality and Protein Digestibility of Vegetable Creams Reformulated with Microalgae Inclusion
Source: Foods. 2023 Jun 16;12(12):2395. doi: 10.3390/foods12122395 (PMC10297650; doi:10.3390/foods12122395)
Supplement: Supplementary file 1 [file foods-12-02395-s001.zip › foods-2445050-supplementary.pdf]

Table S1: Chemical composition of microalgae powders (g/ 100g).

Microalgae	Fat	Saturated fatty acids	Carbohydrates	Sugars	Dietary fibers	Protein	Salt
A. platensis	7.70	2.70	20.30	3.10	3.20	57.50	0.50
C. vulgaris	7.00	2.45	37.80	4.00	20.00	26.30	0.30
T. chui	7.90	7.63	15.50	4.30	11.20	45.26	1.40
N. oceanica	15.10	14.75	17.00	0.09	14.20	45.00	8.00

Table S2: Chemical composition of vegetable creams (g/100 g).

	STD	SP1.5	SP3	CV1.5	CV3	TC1.5	TC3	NO1.5	NO3
**Fat**	3.08	3.17	3.22	3.15	3.21	3.16	3.23	3.23	3.37
**Of which saturated**	0.36	0.39	0.42	0.39	0.41	0.44	0.52	0.51	0.66
**Carbohydrates**	3.75	3.91	4.05	4.28	4.81	3.85	3.95	3.87	3.98
**Of which sugars**	1.24	1.25	1.27	1.27	1.29	1.27	1.29	1.23	1.21
**Protein**	2.79	3.33	3.45	2.66	2.55	2.95	3.6	2.98	2.79
**Salt**	0.55	0.55	0.56	0.55	0.55	0.56	0.58	0.63	0.71

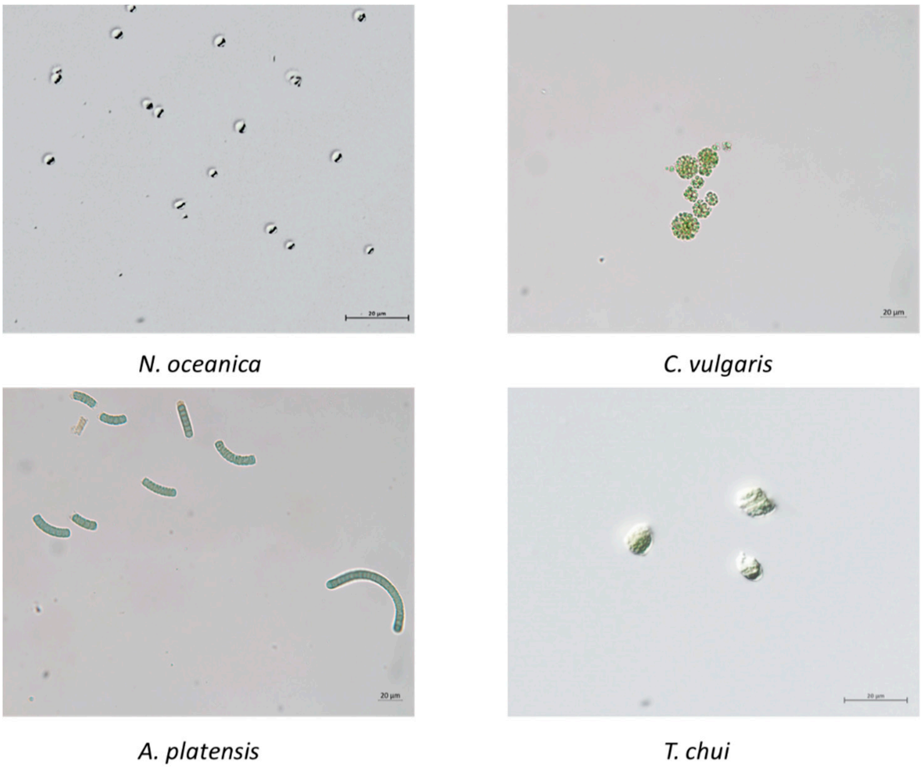

Figure S1. Microscopic images of microalgae single cell ingredients. Photos by M. Soares and B. Schmid.

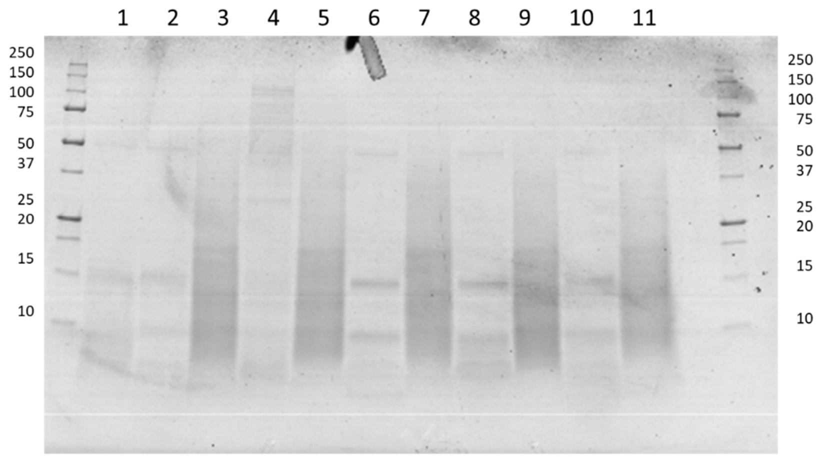

1. Digestion blank 1
2. Spinach cream without microalgae 1 (digested)
3. Spinach cream without microalgae 1 (control)
4. Spinach cream with *Chlorella vulgaris* 1.5% 1 (digested)
5. Spinach cream with *Chlorella vulgaris* 1.5% 1 (control)
6. Spinach cream with *Nannochloropsis oceanica* 1.5% (digested)
7. Spinach cream with *Nannochloropsis oceanica* 1.5% (control)
8. Spinach cream with *Arthrospira platensis* 1.5% 1 (digested)
9. Spinach cream with *Arthrospira platensis* 1.5% 1 (control)
10. Spinach cream with *Tetraselmis chui* 1.5% 1 (digested)
11. Spinach cream with *Tetraselmis chui* 1.5% 1 (digested)

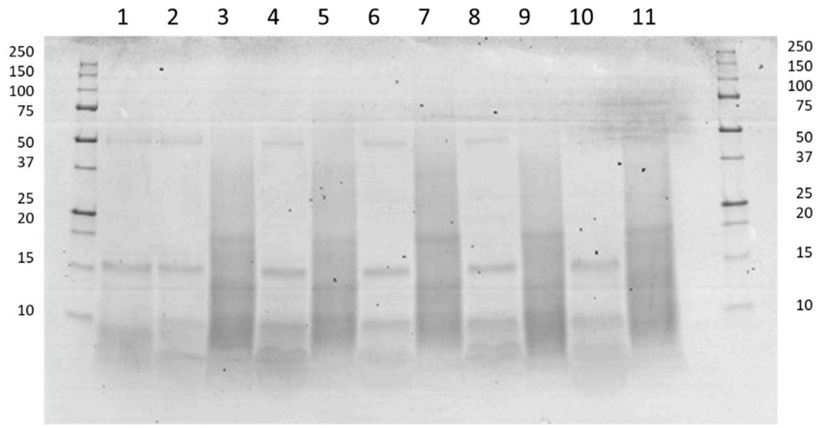

1. Digestion blank 1
2. Spinach cream without microalgae 1 (digested)
3. Spinach cream without microalgae 1 (control)
4. Spinach cream with *Chlorella vulgaris* 3% 1 (digested)
5. Spinach cream with *Chlorella vulgaris* 3% 1 (control)
6. Spinach cream with *Nannochloropsis oceanica* 3% (digested)
7. Spinach cream with *Nannochloropsis oceanica* 3% (control)
8. Spinach cream with *Arthrospira platensis* 3% 1 (digested)
9. Spinach cream with *Arthrospira platensis* 3% 1 (control)
10. Spinach cream with *Tetraselmis chui* 3% 1 (digested)
11. Spinach cream with *Tetraselmis chui* 3% 1 (control)

Figure S2. SDS-PAGE of spinach creams subjected to simulated gastrointestinal digestion (digested) or not (controls).
